# What still presents urgently to ENT during a pandemic? Experience of an ENT rapid access clinic during the coronavirus disease 2019 pandemic

**DOI:** 10.1017/S0022215121000128

**Published:** 2021-01-07

**Authors:** T J Stubington, B Morrison, C Sevilla, O Judd

**Affiliations:** Department of Otorhinolaryngology – Head and Neck Surgery, Royal Derby Hospital, Derby, UK

**Keywords:** COVID-19, Telephone

## Abstract

**Objectives:**

This study sought to determine the conditions that still present to ENT despite government advice to avoid unnecessary travel. It also assessed the impact of social distancing on pathologies presenting to ENT and reviewed the usefulness of telephone consultations in semi-urgent presentations.

**Method:**

A retrospective review was conducted of 97 instances of patient care carried out in the rapid access ENT clinic at a large district general hospital.

**Results:**

Otitis externa and foreign bodies represented 25 per cent and 13 per cent of consultations respectively. Tonsillitis and peritonsillar abscess were uncommon, representing 6 per cent of total consultations. Telephone appointments represented only 28 per cent of total consultations; however, they appeared to reduce the number of physical appointments required.

**Conclusion:**

Otitis externa and foreign bodies continue to be common during the pandemic. Social distancing measures reduced the number of tonsillitis and quinsy presentations. Telephone consultations are effective for certain urgent presentations to ENT, most noticeably nasal trauma and follow up of non-serious pathologies.

## Introduction

The coronavirus disease 2019 (Covid-19) outbreak has been declared an international pandemic. It is thought that Covid-19 is spread via respiratory droplets, necessitating the use of personal protective equipment (PPE) when assessing and managing suspected cases.^1^ The nature of ENT examinations may put practitioners at high risk of transmission,^2^ and this has led ENT UK to release specific guidelines.^3^ Where possible, face-to-face consultations are minimised and telephone consultations are used. Where this cannot be achieved, appropriate PPE is essential.^4^

Prior to the Covid-19 pandemic, a rapid access triage clinic was run by the junior ENT doctor via a ‘bleep’ referral system. With the advent of Covid-19, the ENT rapid access clinic was streamlined to enable review by a senior clinician, in order to facilitate treatment and discharge in as many cases as possible. The intention was to reduce admission rates and potential exposure to coronavirus-positive in-patients in the main hospital. This initial report presents data from the first five weeks of the pandemic, demonstrating the range of conditions that are still presenting to ENT on a semi-urgent basis. We also discuss the patients who require repeat consultations, despite the transmission risk associated with multiple attendances.

## Materials and methods

The emergency clinic online booking calendar was accessed, and all bookings were reviewed over a five-week period from 23rd March to 24th April. The patients’ presenting complaint and history were obtained from the booking system, and further demographic information was obtained from the hospital's electronic records system. Where possible, outcomes were obtained either from Clinic letters or the electronic records system. However, because of Covid-19 and the pressures on the audit department, patient records contained exclusively in the paper case notes could not be retrieved. Data analysis was carried out in Microsoft Excel® spreadsheet software, and figures were also produced in Excel.

## Results

This audit included a total of 97 appointments during a five-week period.

### Indications for consultation

**Fig. 1.**
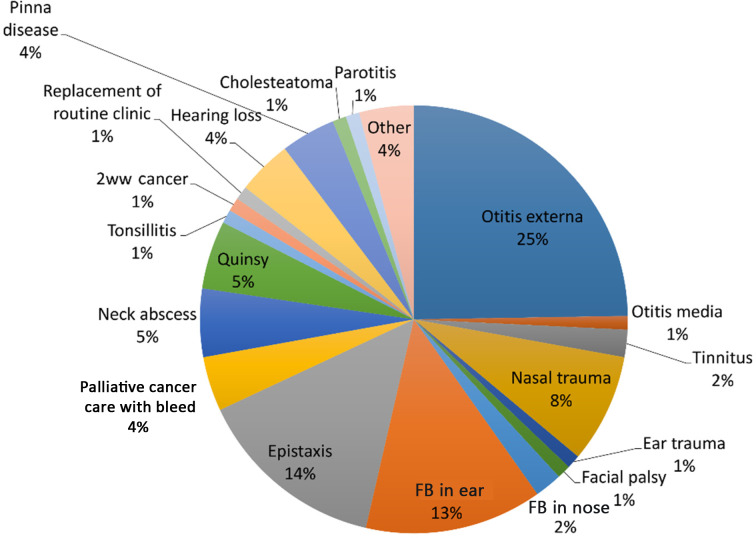
Frequency of each diagnosis seen in clinic by individual appointment. The chart includes repeat attendances for the same pathology as separate data points. 2ww cancer = two-week wait referral for cancer; FB = foreign body

Figure 1 shows the indications for each individual consultation, including re-attendances by the same patient. Otitis externa was by far the most frequent indication for an appointment, followed by epistaxis and foreign bodies in the ear. Surprisingly, patients whose appointments had been cancelled or postponed because of the pandemic made up only 1 per cent of consultations.

### Repeat consultations

Repeat attendances accounted for 26 out of 97 episodes of patient care. The most frequent diagnoses, when the data were adjusted to exclude re-attenders, were: foreign bodies (18 per cent), epistaxis (17 per cent) and otitis externa (15 per cent). Of these re-attendances, 13 were planned repeat consultations arranged by the ENT department, 11 were either arranged via the emergency department or a repeat general practitioner referral, and in 2 cases the patient called the department.

The greatest number of repeat appointments was five in an otitis externa patient; this patient had been through a number of unsuccessful treatments prior to presenting to ENT and was quite resistant to further advice. Aside from this, all remaining patients had either two or three appointments, and 9 of the 16 patients had at least one telephone appointment.

**Fig. 2.**
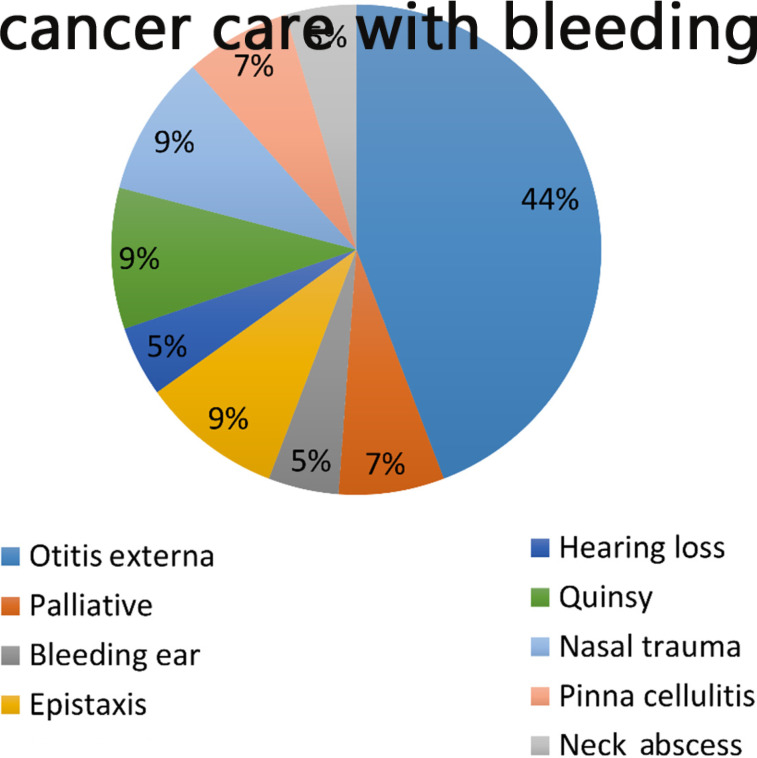
Frequency of each diagnosis in appointments made for a repeat attendance with the same problem.

Overwhelmingly, otitis externa was the most common diagnosis in patients with repeat appointments, followed by epistaxis, nasal trauma and bleeding ears (Figure 2).

### Age distribution

**Fig. 3.**
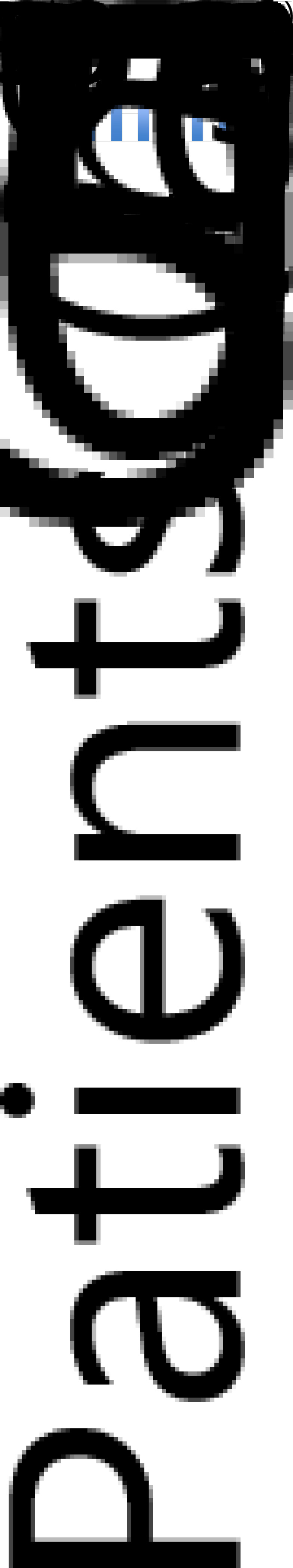
Age distribution in patients presenting to the rapid access clinic.

The most frequently attending age group (Figure 3) was 50–59 years, primarily because of external ear infections. Accounting for repeat appointments did not have a significant effect on age distribution.

### Telephone consultations

As described in Table 1, 26 out of 97 appointments were carried out over the telephone. A total of 13 patients were solely managed via telephone consultations.

**Table 1.** Breakdown of patients managed by telephone consultation, including indication for appointment

Appointment type	Appointments (*n*)	Patients (*n*)	Indications for appointment*
Telephone only	15	13	3 epistaxis, 1 cholesteatoma, 3 nasal trauma, 1 palliative cancer care with bleeding, 1 tonsillitis, 3 ear pain (in 1 patient), 1 peritonsillar abscess, 1 uvula haematoma, 1 Bell's palsy
Clinic converted to telephone	8	6	2 peritonsillar abscess, 1 ear pain, 1 palliative cancer care (2 clinic & 1 telephone), 1 otitis externa, 1 difficult otitis externa (3 clinic & 3 telephone)
Telephone converted to clinic	3	3	2 otitis externa, 1 nasal trauma

* Unless otherwise specified, the numbers represent the number of different patients with the pathology (i.e. ‘3 epistaxis’ reflects three telephone appointments for three different patients with epistaxis)

## Discussion

On the 23rd of March 2020, the UK announced social isolation steps to reduce the spread of coronavirus. These steps, hitherto referred to as ‘lockdown’, brought about dramatic social changes. This dramatic change in the lifestyle of the British public may help to explain some of the trends emerging within the patients seen in our emergency clinic.

### Indication for consultation

Otitis externa was a frequent indication, despite typically being mild and self-limiting. The majority of affected patients had undergone prior telephone appointments with their general practitioner who had prescribed oral antibiotics. Despite the high proportion of otitis externa cases in the clinic, this is likely still less than what might be expected. Using a 1.3 per cent annual incidence and a 3 per cent referral rate to ENT,^5^ one might expect around 23 cases over a five-week period (based on a UK population of 66,435,600 and the Royal Derby Hospital serving a population of 600,000). Accepting the extrapolations inherent in this calculation, one possible explanation for this is that reduced water exposure as a result of swimming pool and beach closures has resulted in fewer cases.

Presentations with foreign bodies and nasal trauma are also potentially explained by social restrictions. Foreign body presentations were mainly in children, likely a consequence of frustration associated with being stuck indoors. We anticipated that reduced car travel and opportunities for sports would reduce nasal traumas. Sadly, lockdown measures around the world have led to a rise in domestic violence cases.^6^ Additionally, with elderly patients being advised to self-isolate, they may not have the level of support to mobilise that they usually would, predisposing them to falls and injuries.

Peritonsillar abscess and tonsillitis represented only 6 per cent of cases. Social distancing has likely impacted the spread of upper respiratory tract infections. The 2002 UK national audit quoted 30 peritonsillar abscesses as the average number treated per year,^7^ which would make 3 cases over a five-week period appropriate. However, previously published data from the Royal Derby Hospital demonstrate a rate of 43 over a period of nine months.^8^ Extrapolating this, five to six cases over a period of five weeks could be expected.

One group of patients with a surprisingly low frequency of attendance were those who had out-patient appointments cancelled or rescheduled. We expected that large numbers of patients with postponed appointments would present via their general practitioners, but so far this has not been the case. This will likely increase as patients’ symptoms become more troublesome.

### Age distribution

The high proportion of children was almost exclusively due to foreign bodies. In the UK, those aged over 70 years have been advised to self-isolate completely. It is interesting to see that compliance with these measures is reflected in the low proportion of patients within this age group presenting to the emergency clinic. The high attendance of those aged 50–59 years is more difficult to explain. Individuals in this age group, though likely still working, may well be assisting older relatives, but are in an age group prone to some co-morbidities. It is possible that because they are less isolated than older populations, they are more willing to present to their general practitioners and hence more likely to be referred.

### Utility of telephone appointments

A key recommendation in the ENT UK guidelines for Covid-19 is to implement telephone-based consultations where practical.^3,9^ A potential concern was that the need for examination to reach a diagnosis for many ENT pathologies may limit the usefulness of telephone consultations. This dataset suggests that, for certain pathologies, telephone consultations can be highly effective. In some cases of epistaxis and nasal trauma, a telephone call was the only appointment required.

• This novel study investigated changes in non-coronavirus disease 2019 (Covid-19) related ENT presentations over the UK ‘lockdown’ period
• The pandemic has resulted in significant changes to health service care provision
• Reductions in face-to-face consultations and use of telephone consultations have been advocated to reduce Covid-19 risk
• Movement and socialising restrictions have likely affected the prevalence of conditions communicated by close proximity
• Despite advice to limit movement and avoid hospital visits, ENT pathologies such as otitis externa and nasal fractures remained common

We have changed our practice such that patients with nasal trauma are primarily managed over the telephone. They are telephoned at 7 days post-injury, and are only brought in for manipulation if they have ongoing concerns regarding appearance or obstruction. In epistaxis cases, minor bleeds with no risk factors or cases with a clear precipitating trauma are managed over the telephone.

Telephone consultations were also effective for checking treatment efficacy in patients managed in the emergency department. Peritonsillar abscesses and certain cases of otitis externa were ideal conditions for this. Ambulatory management of peritonsillar abscess is well established in the literature^8,10^ and telephone follow up is sufficient, provided the abscess has been adequately drained. Similarly, in clear-cut cases of otitis externa where appropriate treatment has not yet been given, pain and discharge were used as measures of improvement assessed over the telephone.

## Conclusion

The data from our emergency clinic have demonstrated that telephone appointments and follow up can be effective for acute presentations to ENT. Certain pathologies such as nasal trauma can be managed effectively via telephone consultation, whereas others, such as otitis externa and foreign bodies, require physical attendance and review by an ENT specialist. In-person appointments may also be necessary for ongoing problems. A surprising finding was the number of patients presenting with otitis externa. However, many of these had received oral antibiotics prior to presentation to ENT, which had not been effective to treat the condition. As a result, presentations were more severe and required microsuction, which can only be carried out in person within an ENT clinic.
